# Investigation of ferroptosis-associated molecular subtypes and immunological characteristics in lupus nephritis based on artificial neural network learning

**DOI:** 10.1186/s13075-024-03356-z

**Published:** 2024-07-03

**Authors:** Li Zhang, Qing Yan, Miao Lin, Juanjuan He, Jie Tian, Zhihan Chen, Fuyuan Hong

**Affiliations:** 1 Department of Nephrology, Shengli Clinical Medical College of Fujian Medical University, Fujian Provincial Hospital, Fuzhou, Fujian PR China; 2 Department of Rheumatology and Immunology, Shengli Clinical Medical College of Fujian Medical University, Fujian Provincial Hospital, 134 Dongjie Road, Fuzhou, 350001 Fujian Province China

**Keywords:** Lupus nephritis, Ferroptosis, Molecular subtype, Immune, Machine learning, Protective model

## Abstract

**Background:**

Lupus nephritis (LN) is a severe complication of systemic lupus erythematosus (SLE) with poor treatment outcomes. The role and underlying mechanisms of ferroptosis in LN remain largely unknown. We aimed to explore ferroptosis-related molecular subtypes and assess their prognostic value in LN patients.

**Methods:**

Molecular subtypes were classified on the basis of differentially expressed ferroptosis-related genes (FRGs) via the Consensus ClusterPlus package. The enriched functions and pathways, immune infiltrating levels, immune scores, and immune checkpoints were compared between the subgroups. A scoring algorithm based on the subtype-specific feature genes identified by artificial neural network machine learning, referred to as the NeuraLN, was established, and its immunological features, clinical value, and predictive value were evaluated in patients with LN. Finally, immunohistochemical analysis was performed to validate the expression and role of feature genes in glomerular tissues from LN patients and controls.

**Results:**

A total of 10 differentially expressed FRGs were identified, most of which showed significant correlation. Based on the 10 FRGs, LN patients were classified into two ferroptosis subtypes, which exhibited significant differences in immune cell abundances, immune scores, and immune checkpoint expression. A NeuraLN-related protective model was established based on nine subtype-specific genes, and it exhibited a robustly predictive value in LN. The nomogram and calibration curves demonstrated the clinical benefits of the protective model. The high-NeuraLN group was closely associated with immune activation. Clinical specimens demonstrated the alterations of ALB, BHMT, GAMT, GSTA1, and HAO2 were in accordance with bioinformatics analysis results, GSTA1 and BHMT were negatively correlated with the severity of LN.

**Conclusion:**

The classification of ferroptosis subtypes and the establishment of a protective model may form a foundation for the personalized treatment of LN patients.

**Supplementary Information:**

The online version contains supplementary material available at 10.1186/s13075-024-03356-z.

## Background

SLE is a chronic autoimmune disease involving multiple organs, characterized by intolerance to autonomic antigens, lymphoid hyperplasia, the production of autologous polyclonal antibodies, immune complex disease, and various tissue inflammation [1, 2]. Lupus nephritis (LN) is a critical complication of SLE and a vital risk factor for mortality in SLE patients. It has been reported that approximately 10–20% of patients with LN will develop end-stage renal disease (ESRD) within 5 years of diagnosis [3, 4]. The current treatment strategy targeting lupus nephritis is mainly based on immunomodulation. However, lack of understanding of the molecular mechanisms underlying LN has hindered the application and development of specific targeted therapies for this progressive disease. Moreover, the complexity of the pathophysiology and genetic diversity result in a significant proportion of patients not responding to the current targeted therapies. Therefore, there is an urgency to explore novel molecular subtypes for early diagnosis and individualized therapy of LN patients.

Ferroptosis is a novel form of iron-catalyzed lipid peroxidation-induced cell death characterized by the disruption of the lipid repair system involving glutathione and GPX4 synthesis [5]. With the continuous expansion and deepening of research, a growing number of genes and signaling pathways that regulate ferroptosis have been gradually discovered [6–8]. Accumulating evidence suggests that ferroptosis is closely related to the onset and progression of diverse kidney diseases, including acute kidney injury (AKI) and chronic kidney disease (CKD) [9–12], revealing that targeting ferroptosis might be a hopeful therapeutic strategy for kidney diseases. On the basis of bioinformatics analysis, the latest study has determined several ferroptosis-related biomarkers as inhibitors or drivers during the progression of LN [13]. Moreover, another study demonstrates that iron imbalance in the proximal tubules enables to the promotion the accumulation of lipid hydroperoxides, and these iron-catalyzed oxidants can subsequently activate protein-and autoantibody-induced inflammatory transcription factors, ultimately leading to the production of stromal cells and cytokine/chemokine, and enhanced immune cell infiltration levels [14]. However, little is known about the molecular subtypes based on ferroptosis and the molecular mechanisms behind how ferroptosis causes kidney damage in LN patients.

The current study systematically evaluated the differentially expressed ferroptosis-related genes (FRGs) in LN and clarified the correlation between the FRG signature and the immune microenvironment of LN patients. Two ferroptosis subtypes were classified on the basis of the expression profiles of the 10 differentially expressed FRGs. Subsequently, we established a 9-gene protective model based on the artificial neural network machine learning, aiming to provide innovative ideas for precise diagnosis and individualized therapy of LN patients at the gene level. Finally, we performed immunohistochemical (IHC) analysis to validate the role of ferroptosis subtype-specific protective genes in patients with LN.

## Methods

### Data download and pre-processing

Two LN-related microarray datasets (GSE32591, GSE127797) were downloaded from the Gene Expression Omnibus (GEO) website database using the R package “GEOquery” [15]. The GSE32591 dataset (GPL14663 platform) contains a total of 93 samples (14 healthy and 32 LN glomeruli tissues vs. 15 healthy and 32 LN tubulointerstitium tissues). The GSE127797 dataset (GPL24299 platform) contains 42 LN glomeruli tissues and 46 LN tubulointerstitium tissues. Batch removal of the original glomerular and tubular gene expression profiles in these two datasets was performed using the ComBat function on the basis of R package of “sva” [16]. Subsequently, a total of 14 healthy and 74 LN glomeruli tissues were utilized to conduct further analysis, and 15 healthy and 78 LN tubulointerstitium tissues were applied for verifying the repeatability of ferroptosis-related molecular subtypes. The analysis of differential expression was conducted using the R package of “limma” [17]. The the screening criteria was |log2 (fold change)|> 1 and adjusted *p*-value < 0.05.

### Unsupervised clustering for ferroptosis-related genes

Initially, 259 FRGs were downloaded from the FerrDb website database (http://www.zhounan.org/ferrdb/). Afterward, 10 differentially expressed FRGs related to LN were selected based on the above screening criteria. On the basis of 10 FRGs expression profiles, 74 LN glomeruli tissues were classified into different subtypes via the unsupervised clustering method using the R package “ConsensusClusterPlus” [18]. The optimal subtype number was identified using the cumulative distribution function (CDF), CDF delta area curves, and a value of consistent cluster score more than 0.9. The k-means algorithm with 1000 cycles were conducted to confirm the stability of the unsupervised clustering analysis. In addition, LN tubulointerstitium tissues were used for to validate the precision of the clustering.

### Assessment of immune cell infiltration in LN

The CIBERSORT deconvolution algorithm, which is based on the "CIBERSORT" R package, was used to assess the relative proportions of 22 types of immune cells in each LN glomerulus tissue [19]. The CIBERSORT algorithm could obtain the inverse fold product *p*-value for each glomerular sample by the Monte Carlo sampling method using the LM22 signature matrix. The sum of the estimated percentages of the 22 immune cell types in each sample was 100%, and a CIBERSORT *p*-value less than 0.05 was considered significant for immune cell fractions in the sample.

### Evaluation of the correlation between genes and infiltrated immune cells

The Pearson correlation analysis was performed to assess the correlation between differentially expressed FRGs and immune cell properties using the R package of “psych”. Correlation coefficients with a *p*-value less than 0.05 were considered to be significantly correlated. The results of correlation analysis were presented using the R package of “corrplot”.

### GSVA and GSEA analysis

GSVA enrichment analysis, a non-parametric unsupervised algorithm, was utilized to assess the variations of enriched gene sets between distinct ferroptosis subtypes using the “c2.cp.kegg.v7.4.symbols” and “c5.go.bp.v7.5.1. symbols” files downloaded from the MSigDB online database. Subsequently, the R package “limma” was applied for screening enriched pathways and biological functions via comparing GSVA scores between distinct ferroptosis subtypes. The *p*-value < 0.05 and |t value of GSVA score|> 2 were considered to be remarkably enriched.

GSEA is a calculation algorithm to clarify the distribution differences of a priori defined gene set between two groups. The R package of “clusterProfiler” was utilized to identify the significantly enriched pathways and biological functions between high and low NeuraLN group. the “c2.cp.kegg.v7.4.symbols” and “c5.go.bp.v7.5.1. symbols” files were selected as the reference gene lists. A *p*-value less than 0.5 was considered statistically significant.

### Construction of a diagnostic model based on artificial neural network machine learning

The R package “Boruta” was applied for screening important variables associated with LN subtypes. Subsequently, a total of 74 LN glomeruli tissues were selected for constructing the neural network model using the R package “neuralnet” [20] based on the expression profiles of these important variables. The 74 LN glomeruli samples were randomly divided into a training set (70%, *N* = 53) and validation set (30%, *N* = 21). The number of hidden neuron layers were set as two-thirds of the number of the input layer. As a result, eight hidden layers were chosen as the best model parameter for developing a LN classification model based on predicted gene weight information. Subsequently, the constructed neural network model in the training set was verified in the validation set. The "ROSE" R package was used to demonstrate model classification performance and draw the ROC and Precision-Recall (P-R) curves. Finally, the weight values of important genes were utilized to calculate the disease classification score (NeuraLN): Σ_i_ weight values_i_ × Expression level of gene_i_. On the basis of the mean NeuraLN, the 74 LN glomeruli samples were classified into high and low score groups.

### Construction and validation of a nomogram model

The nomogram model containing NeuraLN and immunescore information was built to assess the occurrence of LN subtypes using the R package of “rms”. Each factor has a corresponding score, and the “total score” exhibits the aggregation of the scores of these factors. A calibration curve was applied for estimating the predictive performance of the nomogram model.

### Immunohistochemical staining

Formalin-fixed, paraffin-embedded glomeruli tissues were collected from 13 control and 19 LN patients with LN diagnosed at the Fujian Provincial Hospital. This study was approved by the Fujian Provincial Hospital Ethics Committee. Briefly, samples were fixed in 4% buffered formalin for more than 24 h, embedded in paraffin and placed at room temperature until sectioning. Subsequently, the samples were cut into 2-3um slices and flattened on the warm water of the spreading machine. After being paraffinized with xylene. slices were hydrated using a streptavidin–biotin-peroxidase conjugate and were subjected to immunohistochemical analysis. To determine immunoreactivity, the slices were heating in 0.01 M citrate buffer for antigen repair. After washing, slices were incubated in PBS containing 10% normal goat serum to eliminate nonspecific staining and then incubated with the following primary antibody: rabbit anti-DPYS antibody (1:200, proteintech), rabbit anti-PAH antibody (1:200, proteintech), rabbit anti-HAO2 antibody (1:200, proteintech), rabbit anti-BHMT antibody (1:200, proteintech), rabbit anti-GAMT antibody (1:200, proteintech), rabbit anti-CUBN antibody (1:200, proteintech), rabbit anti-GSTA1 antibody (1:200, proteintech), rabbit anti-SLC27A2 antibody (1:200, proteintech), rabbit anti-ALB antibody (1:200, proteintech) at 4℃ overnight. After washing, these slices were incubated for 120 min at room temperature with HRP-labelled goat anti-rabbit secondary antibody (1:200, Thermo Fisher). Finally, the sections underwent counter-staining with Mayer's hematoxylin and were dehydrated and mounted for further analysis. The images of sections were acquired with a fluorescence microscope with 200 × magnification, and the fluorescence intensity was exhibited by the integrated option density (IOD).

### Statistical analysis

The differences between the two groups were compared using the Wilcoxon test or t test. The correlation tests were performed using the Spearman analysis with the R package “psych”. In comparisons between groups, a value of p less than 0.05 was considered to be remarkably significant. The R software (version: 4.1.0) was applied for data processing.

## Results

### Identification of differentially expressed ferroptosis regulators and assessment of the immune cell infiltration in LN patients

**Fig. 1:**
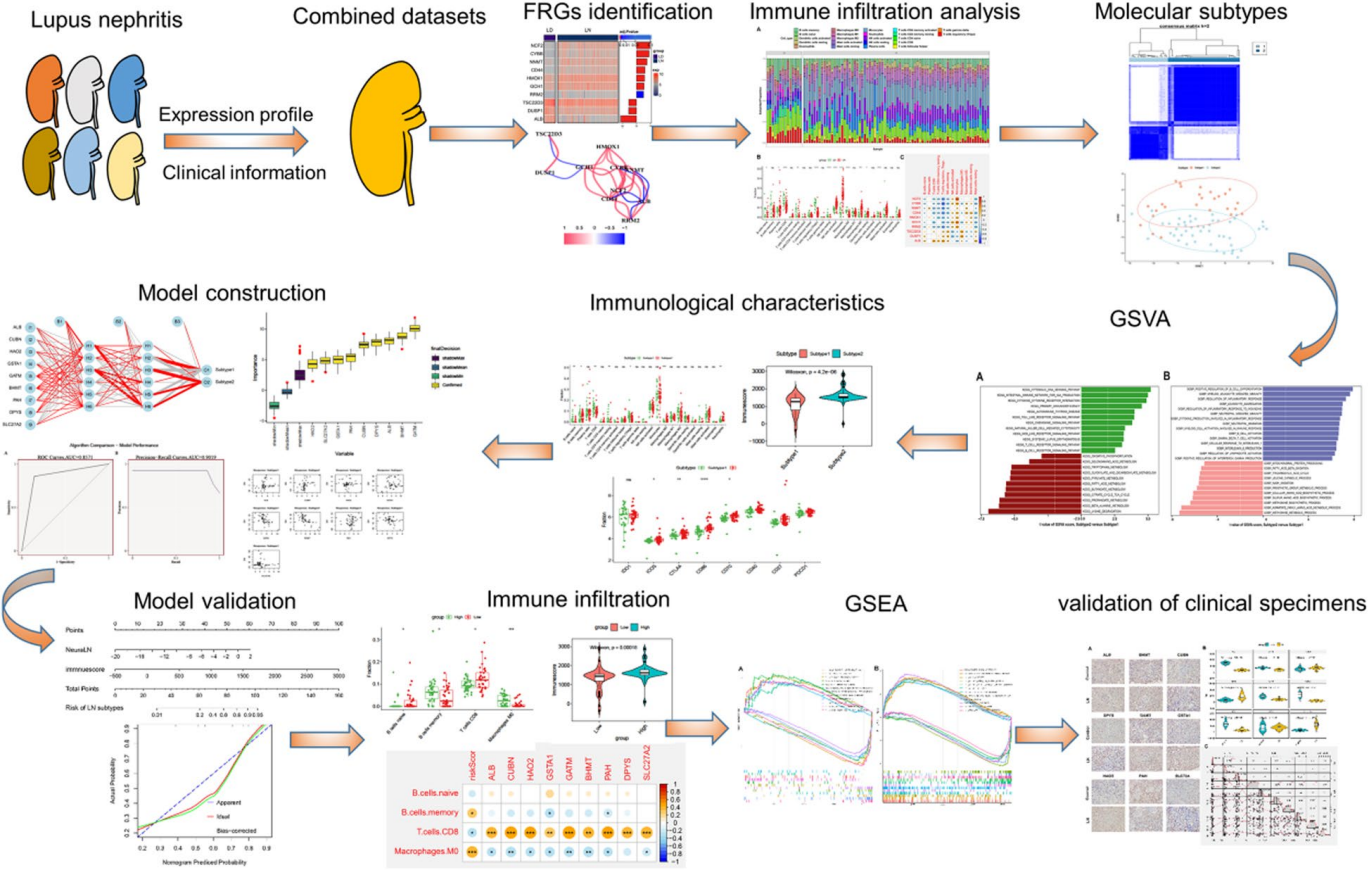
The flow chart of this study

**Fig. 2:**
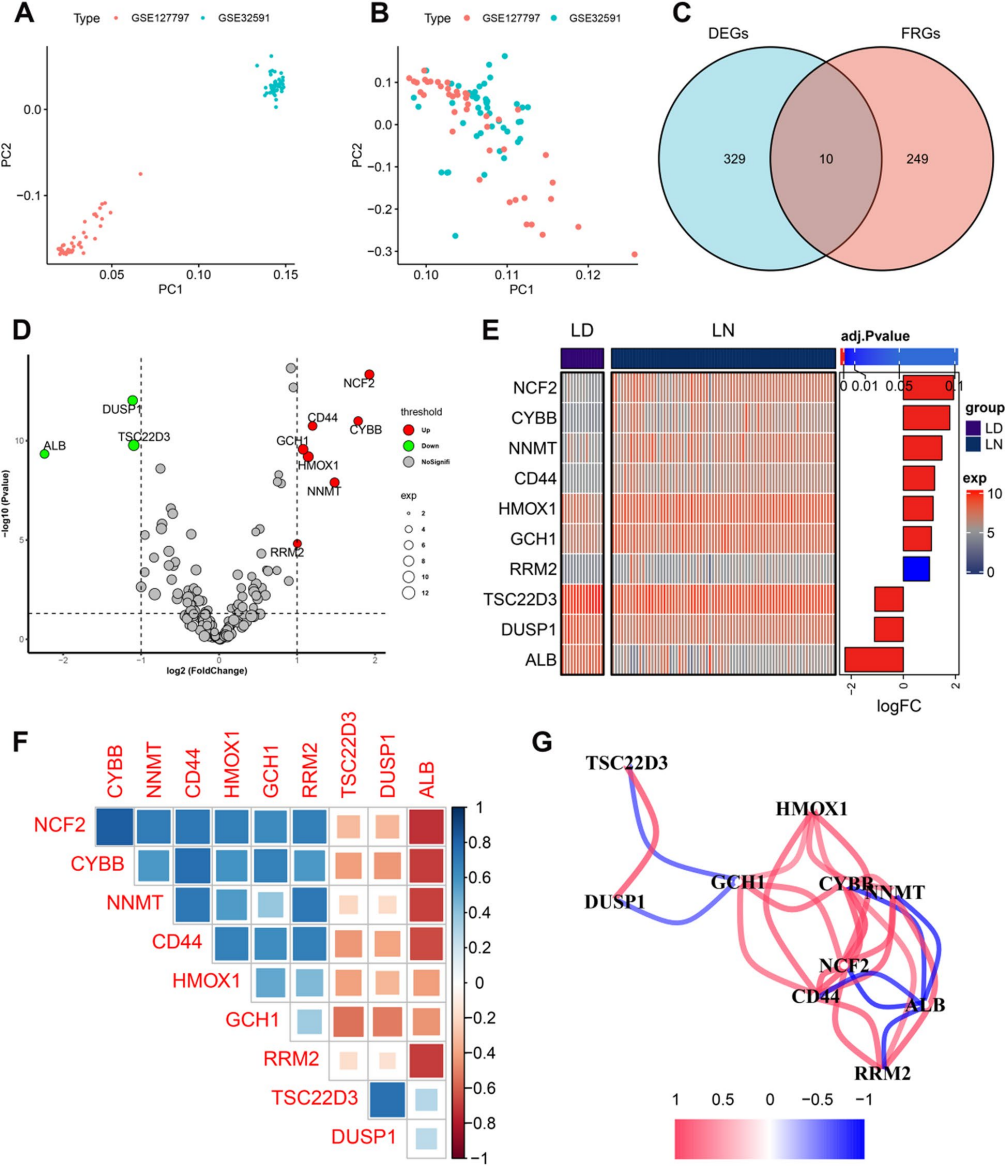
Identification of differential expressed ferroptosis regulators in LN patients. **A**, **B** The PCA plot exhibiting the expression profiles of GSE32591 and GSE127797 before (**A**) and after (**B**) correction of batch effect. **C** Venn diagram exhibiting 10 differentially expressed FRGs in LN patients. **D** Volcano plot depicting the mRNA expression levels of FRGs between healthy individuals and LN patients. **E** Heatmap exhibiting the differentially expressed FRGs between the aforementioned groups. **F**, **G** Correlation plot (**F**) and network diagram (**G**) of 10 differentially expressed FRGs. Positive correlations were marked in blue and negative correlations were marked in red. The size of the rectangle showing the specific value of the correlation coefficients

The whole gene expression landscapes of two GEO datasets (GSE32591 and GSE127797) including 14 healthy (LD) and 74 LN glomeruli tissues, were downloaded from the GEO online database. A detailed flow chart of the current study is presented in Fig. 1. Samples from distinct platforms exhibited significantly different clustering before batch effect correction (Fig. 2A), whereas they clustered together after batch removal (Fig. 2B). To clarify the role of ferroptosis regulators in the progression of LN, we first obtained a total of 339 differentially expressed genes (DEGs) (241 upregulated and 98 downregulated genes) related to LN. When we combined these LN-related DEGs with 259 ferroptosis-related signatures, we eventually identified 10 differentially expressed ferroptosis regulators (Fig. 2C). Among them, the expression levels of NCF2, CD44, CYBB, GCH1, HMOX1, NNMT, and RRM2 genes were remarkably higher, whereas ALB, DUSP1, and TSC22D3 genes exhibited markedly lower expression levels in LN glomeruli tissues than those in LD (Fig. 2D,E). Subsequently, spearman’s correlation analysis between these differentially expressed FRGs was utilized to elucidate whether ferroptosis functioned essentially in LN. Genes with correlation coefficients greater or less than 0.5 were displayed in the gene relationship network graph (Fig. 2F,G), indicating a fairly close association among these differentially expressed FRGs. These results demonstrated that the interactions among FRGs may play a critical role in the progression of LN.

**Fig. 3:**
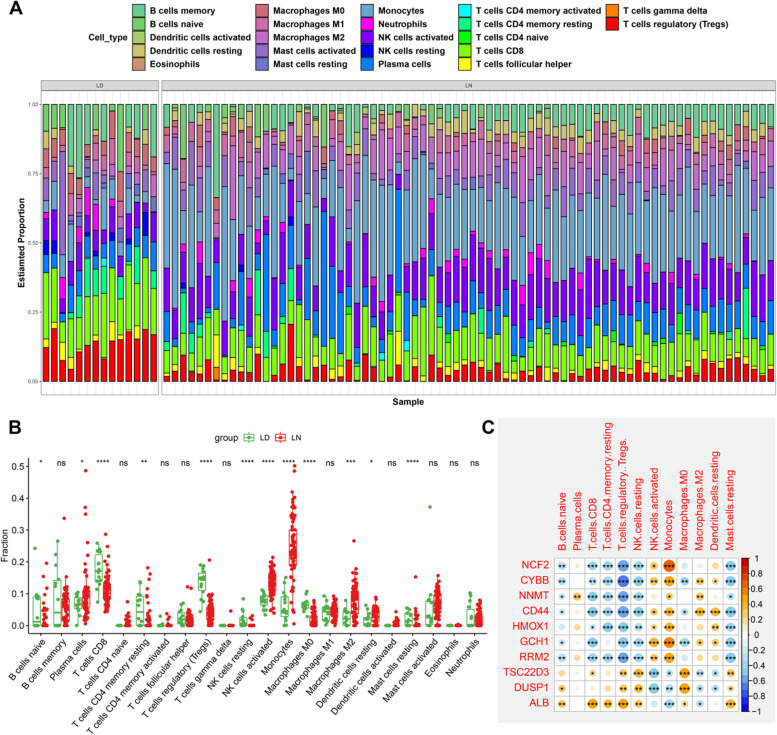
Immunological characteristics of normal subjects and LN patients and correlations between the characteristic FRGs and immune cells. **A** Stack chart exhibiting the relative proportions of 22 infiltrated immune cells from LD and LN samples. **B** Box plots exhibiting the alterations in infiltrated immune cells between LD and LN groups. **C** Heatmap exhibiting the correlations between characteristic FRGs and distinct immune cell compositions. *p < 0.05, **p < 0.01, ***p < 0.001. Positive correlations were marked in red and negative correlations were marked in blue. The size of the circle showing the specific value of the correlation coefficients

To investigate the differences in immune microenvironment between the LN and non-LN glomeruli tissues, we analyzed the percentages of 22 infiltrated immune cells between LN patients and control individuals based on the CIBERSORT algorithm (Fig. 3A). The results revealed that LN patients displayed higher infiltration levels of naïve B cells, plasma cells, activated NK cells, monocytes, M2 macrophages, and resting dendritic cells, suggesting that the progression of LN was clearly accompanied by the alterations in immune response (Fig. 3B). We performed a correlation analysis to further understand the relationship between FRGs and significantly infiltrated immune cells in LN patients and discovered that CD8^+^ T cells, resting memory CD4^+^ T cells, Regulatory T cells (Tregs), resting NK cells, monocytes, and resting mast cells were significantly correlated with these 10 differentially expressed FRGs (Fig. 3C), implying that feroptosis regulators may act as key factors in regulating immune infiltration levels.

### Identification of ferroptosis subtypes in LN

**Fig. 4:**
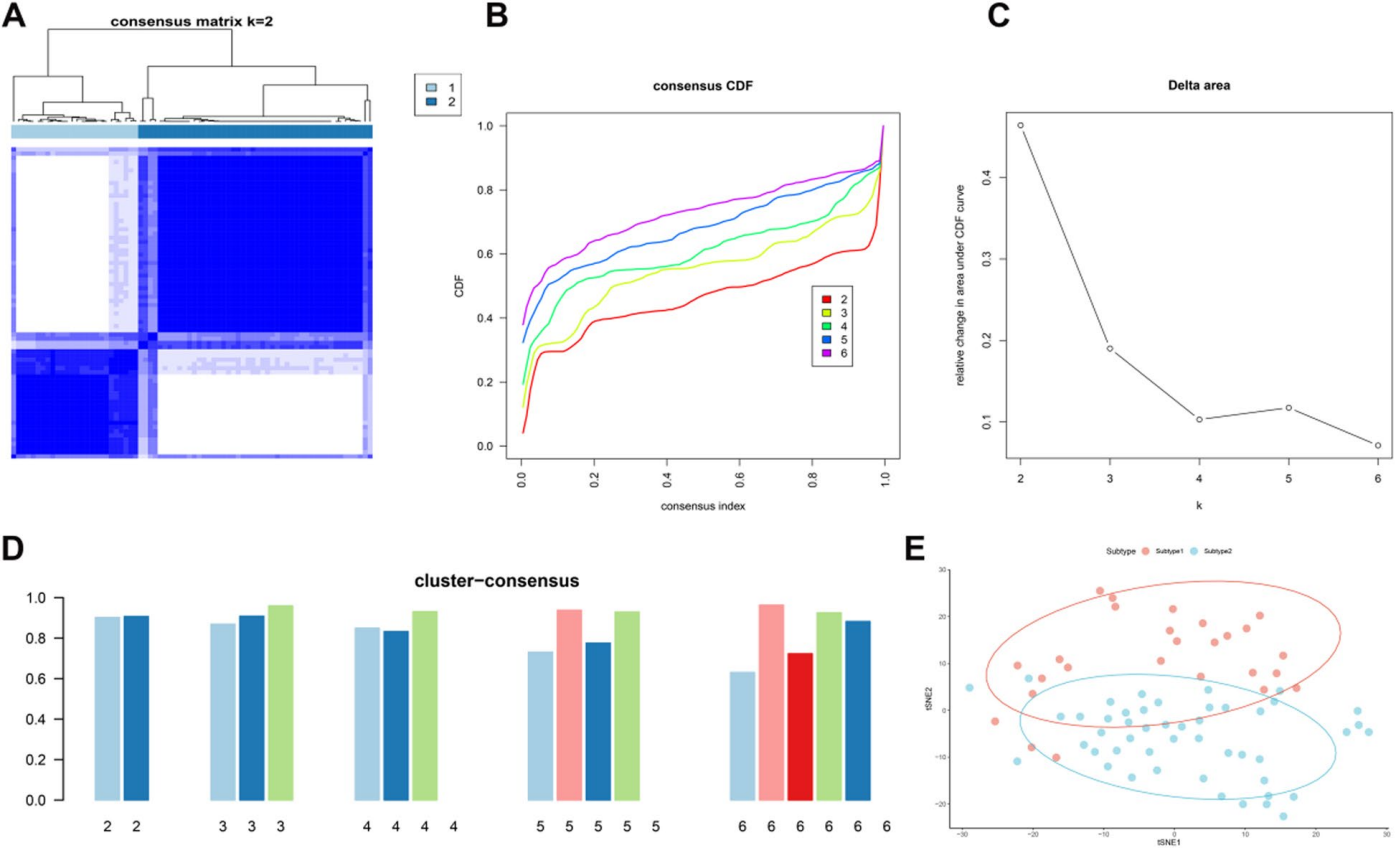
Characterization of molecular subtypes based on characteristic FRGs. **A** Consensus matrix in LN patients (k = 2). Both the rows and columns of the matrix represent samples. The consistency matrix ranges from 0 to 1, from white to dark blue. **B** Consensus CDF when k = 2 to 6. **C** Delta area under CDF curve. **D** Consensus clustering score exhibiting the subtype score when k = 2–6. **E** t-SNE exhibiting that LN patients were classified into two distinct ferroptosis subtypes

To illustrate the expression patterns related to ferroptosis in LN, we grouped the 74 LN glomeruli tissues using unsupervised clustering analysis based on the expression profiles of 10 differentially expressed FRGs. Relatively crisp boundaries were displayed in the consensus clustering matrix when the cluster numbers were set to two (k = 2) (Fig. 4A). In addition, the CDF curves exhibited the relative lower range ability at consensus index 0.2–0.6 when k = 2 (Fig. 4B). The differences in the area under the CDF curves were presented between the two CDF curves (k and k-1) (Fig. 4C), Moreover, the consensus score for each subgroup was more than 0.9 when k = 2 (Fig. 4D). We eventually grouped 74 LN patients into two ferroptosis-related subtypes, including Subtype 1 (*n* = 26) and Subtype 2 (*n* = 48). The analysis of t-Distributed Stochastic Neighbor Embedding (tSNE) also exhibited the distinct clusters between these two subtypes (Fig. 4E). We next utilized the 78 LN tubulointerstitium tissues to verify the repeatability of the clustering. Consistently, two distinct subtypes were also clearly identified (Figure S1A-E), further demonstrating that there are two ferroptosis-related subtypes in LN patients.

### Identification of biological functions of distinct ferroptosis subtypes

**Fig. 5:**
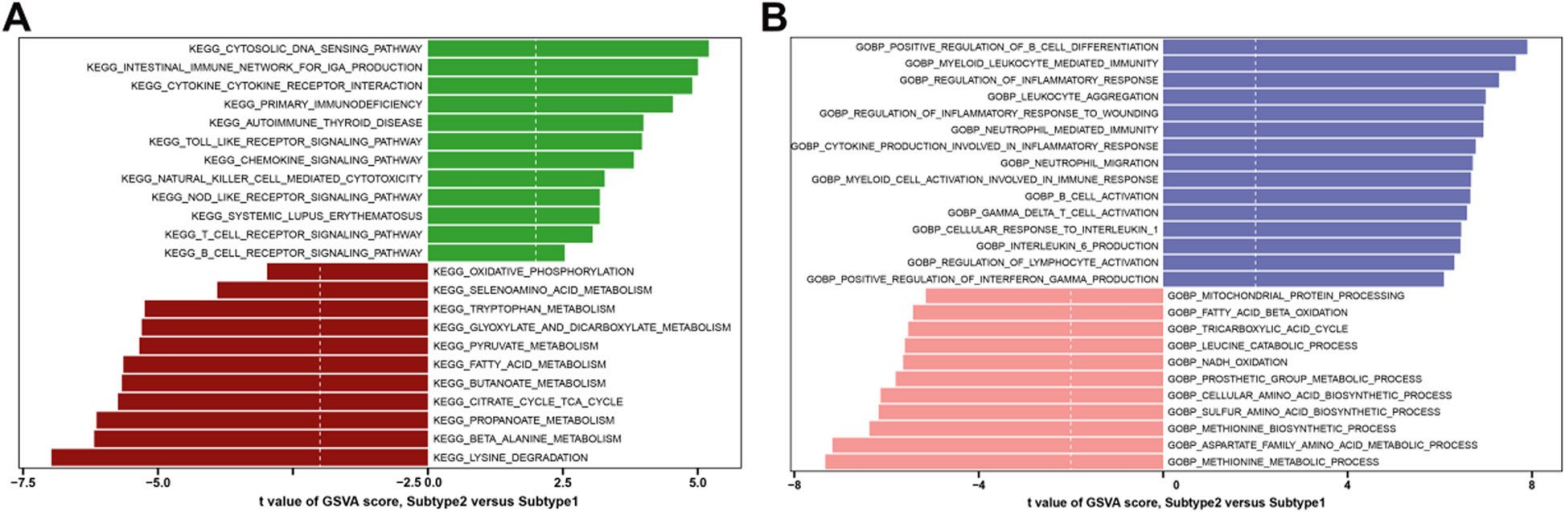
Identification of distinct biological functions and signaling pathways between subtypes. **A**, **B** GSVA exhibiting distinct biological processes (**A**) and signaling pathways (**B**) between subgroups

To clarify the functional differences in different ferroptosis subtypes, we performed GSVA analysis and found that metabolism-related signaling pathway, oxidative phosphorylation, and the TCA cycle were significantly upregulated in Subtype 1, while DNA regulation, chemokine signaling, cytokine receptor interaction, autoimmune diseases, various immune cells pathways such as T cell receptor, B cell receptor, Nod-like receptor, natural killer cells activation, and Toll-like receptor pathway were upregulated in subtype 2(Fig. 5A). Additionally, functional enrichment results indicated that Subtype 1 was remarkably associated with amino acid biosynthesis, fatty acid beta-oxidation, NADH oxidation, TCA cycle, mitochondrial protein processing, and metabolic processes. Immune-related biological functions such as B cell differentiation, neutrophil-mediated immunity, B and T cell activation, gamma delta T cell activation, and disease-related immunity, on the other hand, were significantly enriched in subtype 2. Otherwise, Subtype 2 also enriched in inflammatory responses such as leukocyte aggregation, cytokine production, lymphocyte activation, and IL1 and IL6 production (Fig. 5B). These results revealed that ferroptosis Subtype 2 might be implicated in various immune responses.

### Identification of immune infiltration and immune checkpoint characteristics between ferroptosis subtypes

**Fig. 6:**
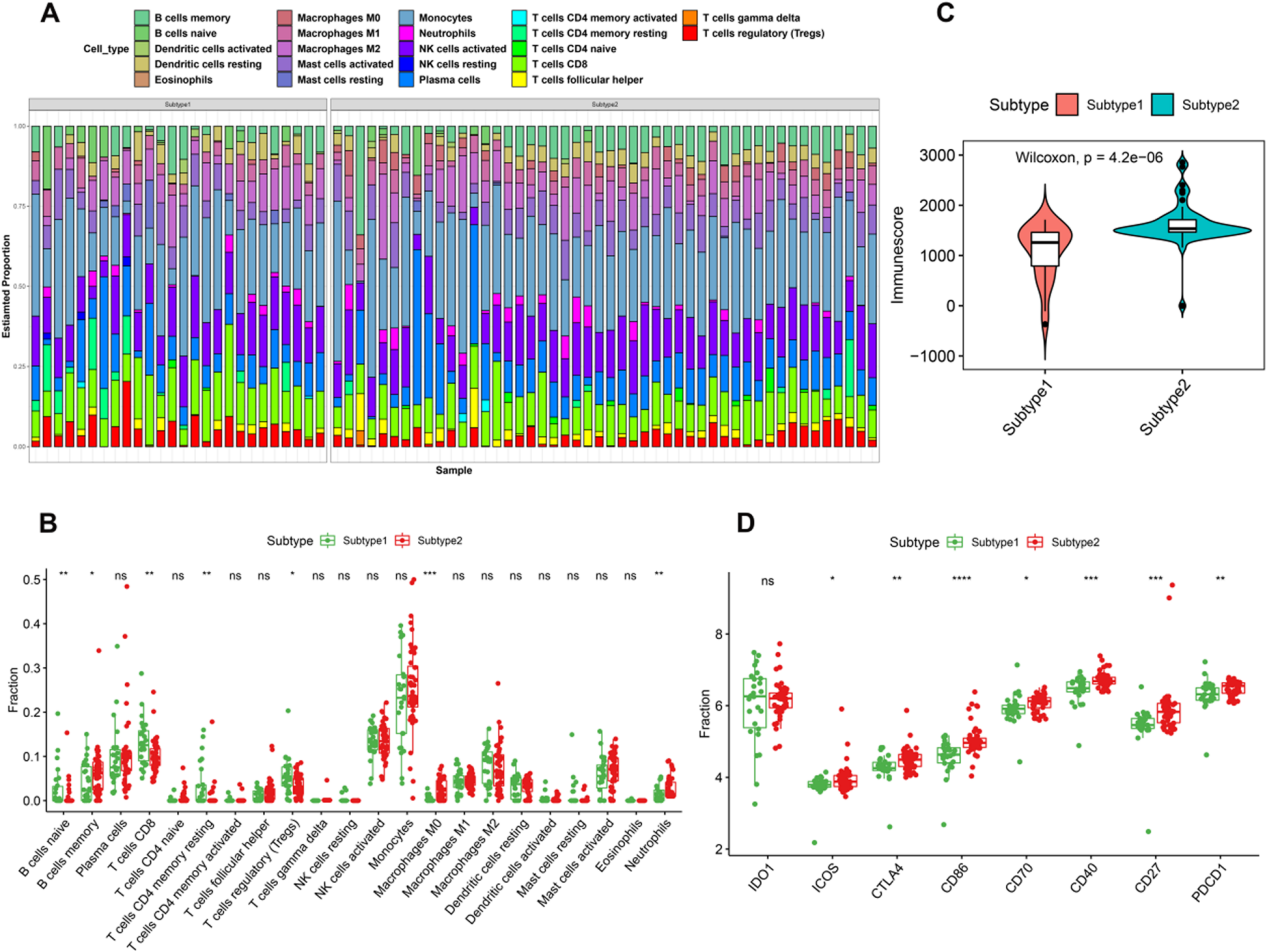
Association between ferroptosis subtypes and immunological features. **A** Stack chart exhibiting the abundances of 22 immune cell subpopulations from Subtype 1 and Subtype 2. **B** Box plots exhibiting the differences in the relative abundance of infiltrated immune cell types between the two ferroptosis subtypes. **C** Box plots exhibiting the differences in the immune score between the two ferroptosis subtypes. **D** Box plots exhibiting the mRNA expression of immune checkpoints between two ferroptosis subtypes

To explore the differences in immune microenvironment between distinct ferroptosis subtypes, we first comprehensively assessed infiltrated immune cells using the CIBERSORT algorithm. The altered immune infiltration levels were found between ferroptosis Subtype 1 and Subtype 2 (Fig. 6A). Subtype 1 exhibited greater proportions of naïve B cells, CD8^+^ T cells, resting memory CD4^+^ T cells, and Tregs, whereas the abundance of memory B cells, M0 macrophages, and neutrophils were markedly higher in Subtype 2 (Fig. 6B). To further elucidate the levels of immune infiltration between the two subtypes, we calculated the immune score using the ESTIMATE algorithm. Consistently, Subtype 2 also exhibited a greater immune score (Fig. 6C), indicating that ferroptosis Subtype 2 had noticeably increased infiltration of immune cells.

Subsequently, we further evaluated the expression levels of classical immune genes and immune checkpoints in LN patients with distinct ferroptosis subtypes. We found the extraordinarily enhanced expression levels of immunosuppression, MHC, and immunostimulatory-related genes in Subtype 2 when compared with Subtype 1 (Figure S2A-C), indicating that ferroptosis Subtype 2 presented stronger immune responses than ferroptosis Subtype 1. Moreover, we also found that the expression of immune checkpoints had the ability to distinguish between ferroptosis subtypes. The expression levels of immune checkpoint inhibitor-related genes (ICOS, CTLA4, CD86, CD70, CD40, CD27, and PDCD1), for example, were significantly higher in ferroptosis subtype 2 than in ferroptosis Subtype 1 (Fig. 6D), indicating that Subtype 2 may be more effective in immunotherapy.

### Construction and evaluation of a predictive model

**Fig. 7:**
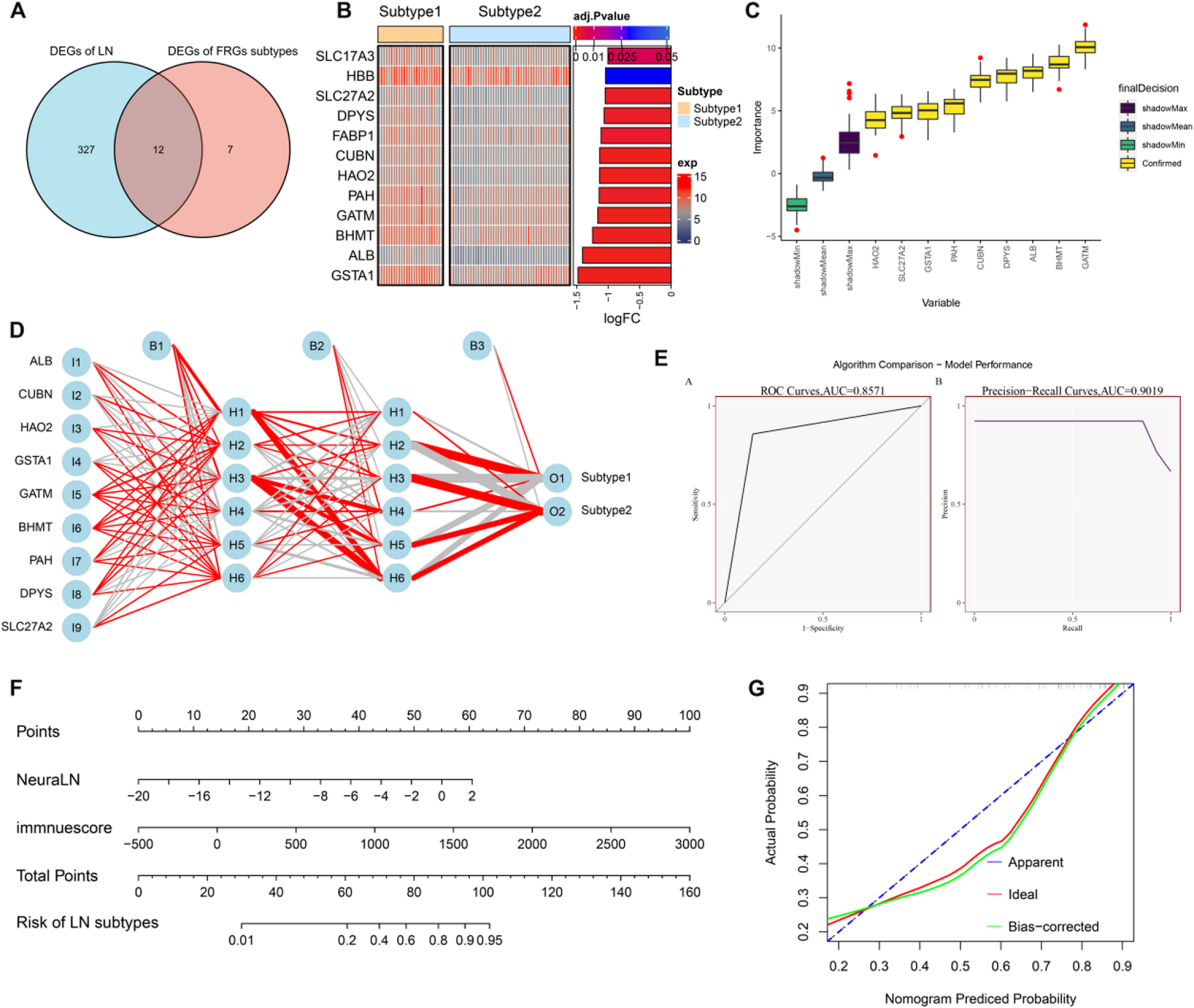
Construction of a protective model for prediction of LN patients. (**A**, **B**) Venn diagram (**A**) and heatmap (**B**) exhibiting 12 ferroptosis subtype-specific feature genes in LN patients. **C** A total of 9 characteristic genes were obtained using the using the Boruta feature selection algorithm. **D** The visualization of the neural network machine learning model. **E** Assessment of the classification performance of the neural network machine learning model in the validation cohort. **F** Representative nomogram based on NeuraLN and immune score for predicting LN progression. **G** Representative calibration curves for assessing the diagnostic accuracy of the nomogram

To further validate the molecular subtypes based on FRGs, we first identified the subtype-specific DGEs by intersecting the DEGs related to LN with the subtype-related DEGs (Fig. 7A). We eventually acquired a total of 12 subtype-specific DEGs, all of which were significantly downregulated in Subtype 2 (Fig. 7B). Subsequently, we identified 9 important variables closely associated with subtypes using the Boruta feature selection algorithm as follows: ALB, BHMT, CUBN, DPYS, GAMT, GSTA1, HAO2, PAH, and SLC27A2 (Fig. 7C). Afterward, the training cohort was utilized to establish an artificial neural network model based on the expression profiles of these 9 important features. According to the output results of the model (Fig. 7D), the intact training was conducted in 2300 steps, and the weight values of the neural network model ranged from -1.62 to 1.08. The weight predictions were as follows: -1.04961 (ALB), − 0.24042 (CUBN), -0.57845 (HAO2), -0.33385 (GSTA1), 0.003948 (GATM), 0.146428 (BHMT), 1.077421 (PAH), 0.042221 (DPYS), and -1.61736 (SLC27A2) (Supplementary Table 1 and Figure S3). In addition, we assessed the classification performance of the neural network model in the validation cohort by calculating the areas under the ROC and Precision-Recall (P-R) curves, and the value was 0.8571 and 0.9019, respectively (Fig. 7E), suggesting that this model was capable of distinguishing the subtypes of LN. Finally, these 9 feature genes were applied for constructing predictive scores (NeuraLN) according to their weight values and expression levels. We also developed a nomogram model for predicting the risk of LN subtypes, and the results suggested that the nomogram had the ability to predict the classification of LN subtypes according to the NeuraLN and immunescore (Fig. 7F). The calibration curves used for the predictive probability demonstrated the accuracy of a nomogram model (Fig. 7G).

**Fig. 8:**
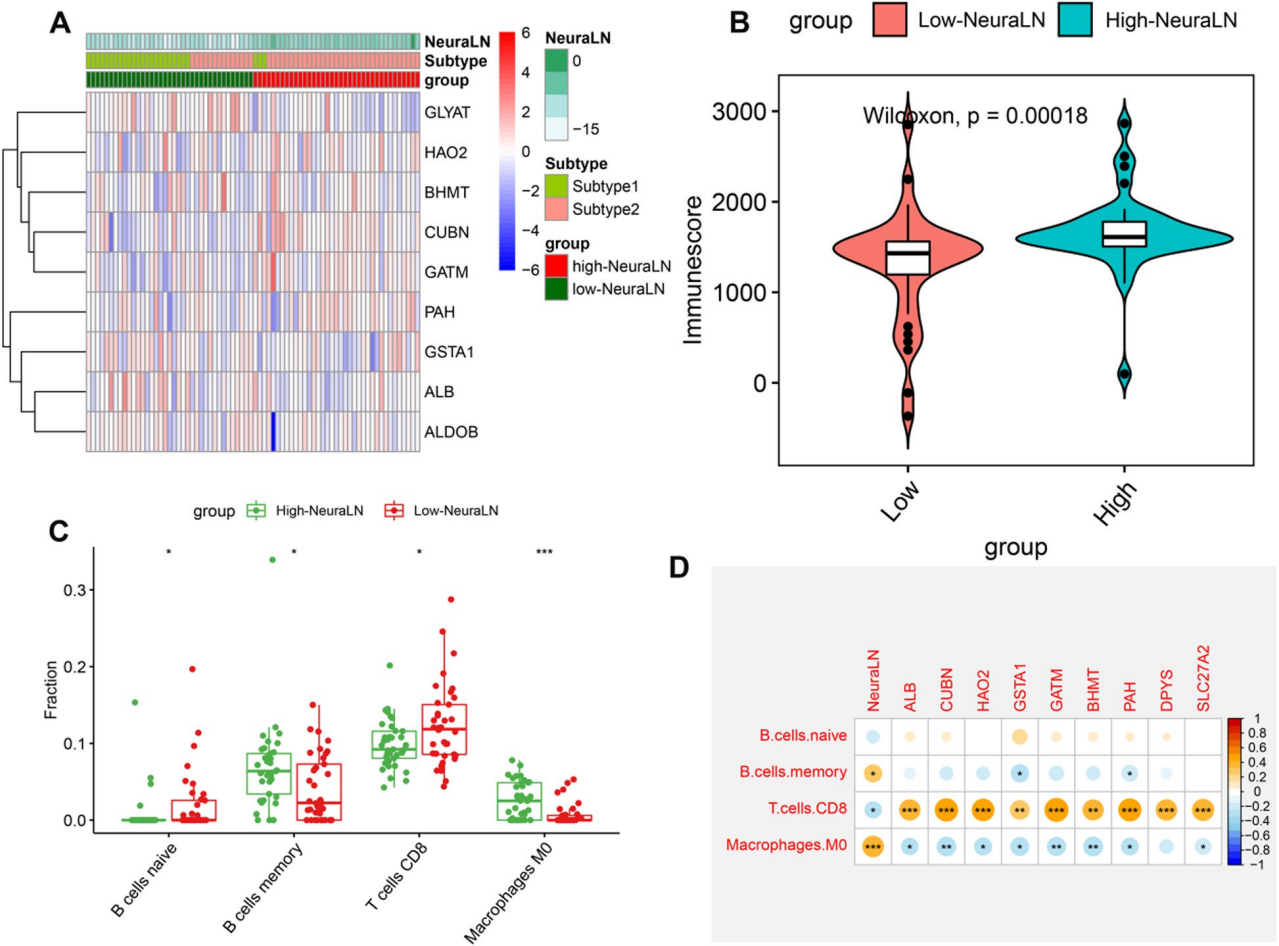
Association between NeuraLN scoring model and immune microenvironment. **A** Heatmap exhibiting the expression profiles of the 9 model genes in the validation cohort. **B** Box plots exhibiting the differences in immune score between the low-and high-NeuraLN group. **C** Box plots exhibiting the differences in the relative abundances of infiltrated immune cell types between the low-and high-NeuraLN group. **D** Heatmap exhibiting the correlations between model genes and distinct immune cell compositions. *p < 0.05, **p < 0.01, ***p < 0.001

Subsequently, we further explored the association between the ferroptosis subtypes and NeuraLN. Subtype 1 was remarkably correlated with low NeuraLN, whereas Subtype 2 was mainly associated with high NeuraLN. The results of the heatmap revealed a significant difference in the expression levels of 9 model genes between the high and low-NeuraLN groups (Fig. 8A). Consistently, the high-NeuraLN group also exhibited a relatively higher immune score (Fig. 8B). These results indicated that the high-NeuraLN group may be closely associated with immunity and may have the ability to predict the ferroptosis subtypes in LN patients. In addition, the abundances of 22 kinds of infiltrated immune cells were further elucidated in the low and high NeuraLN groups. The proportions of infiltrated immune cells such as memory B cells and M0 macrophages were higher in the low NeuraLN group, while high NeuraLN group presented higher infiltration levels of naïve B cells and CD8^+^ T cells (Fig. 8C). Later, we assessed the correlation between these 9 model genes and significantly infiltrated immune cells and found these genes were markedly correlated with CD8^+^ T cells and M0 macrophages (Fig. 8D).

**Fig. 9:**
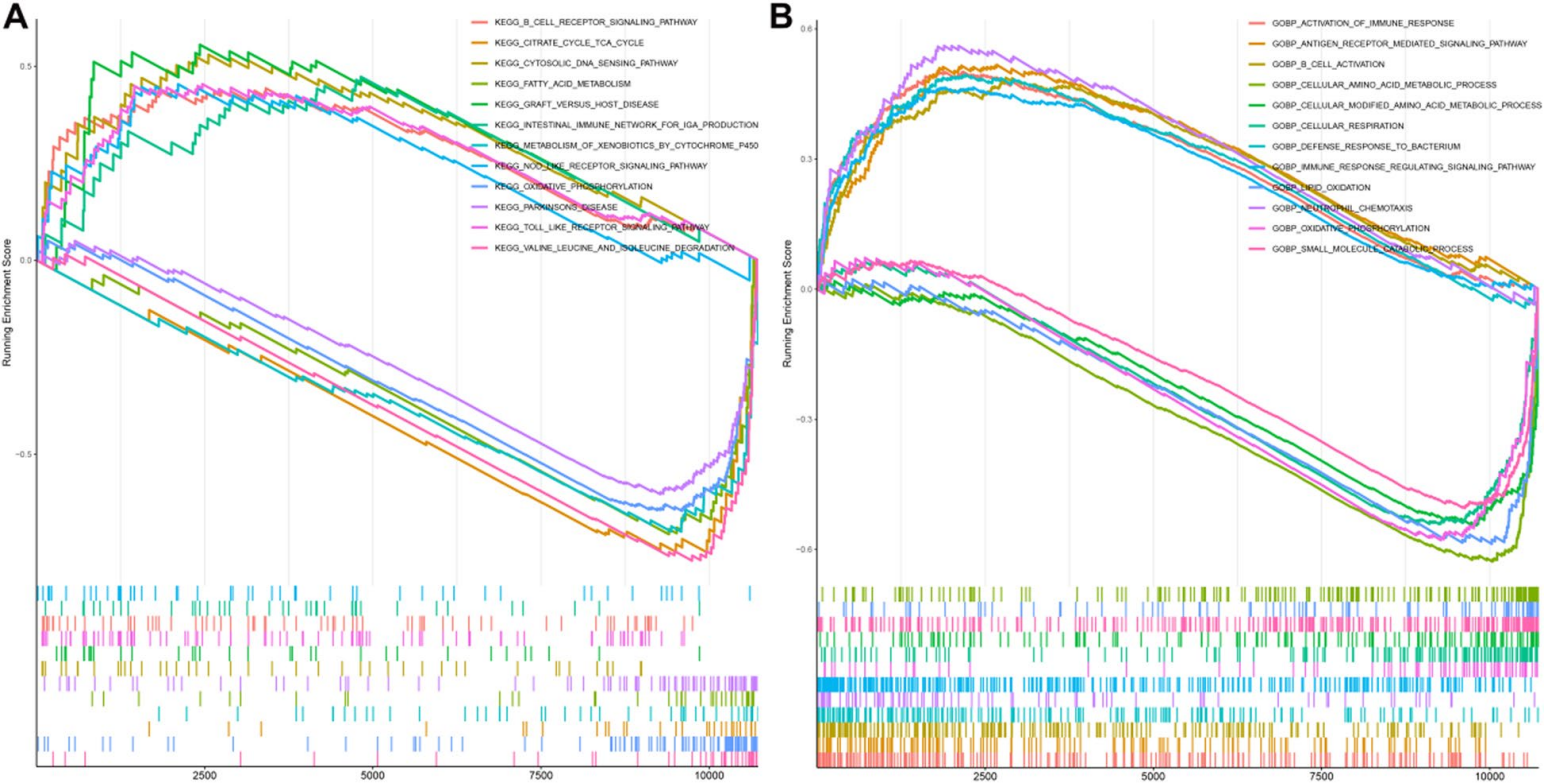
Identification of distinct biological functions and signaling pathways between low-and high-NeuraLN group. **A**, **B** GSEA exhibiting distinct signaling pathways (**A**) and biological processes (**B**) between low-and high-NeuraLN group

To further clarify the functional differences between different NeuraLN groups, we conducted GSEA analysis and found the high NeuraLN group was primarily related to immune-related signaling pathways and biological functions (Fig. 9A,B). The high NeuraLN group was found to be significantly enriched in the following signaling pathways:KEGG_B_CELL_RECEPTOR_SIGNALING,KEGG_INTESTINAL_IMMUNE_NETWORK_FOR_IGA_PRODUCTION,KEGG_NOD_LIKE_RECEPTOR_SIGNALING_PATHWAY,KEGG_TOLL_LIKE_RECEPTOR_SIGNALING_PATHWAY.We have noted these pathways as they are associated with various aspects of immune response and signal transduction. And the high NeuraLN group predominantly demonstrates the following biological functions:ACTIVATION_OF_IMMUNE_RESPONSE,ANTIGEN_RECEPTOR_MEDIATED_SIGNALING_PATHWAY,B_CELL_ACTIVATION,DEFENSE_RESPONSE_TO_BACTERIUM,IMMUNE_RESPONSE_REGULATING_SIGNALING_PATHWAY,NEUTROPHIL_CHEMOTAXIS.These functions highlight the group's significant involvement in the immune response.Combined with these results, it was suggested that the high NeuraLN group was mainly involved in immune activation, and LN patients with this NeuraLN would be more likely to benefit from immunotherapy.

### External validation of clinical specimens

**Fig. 10:**
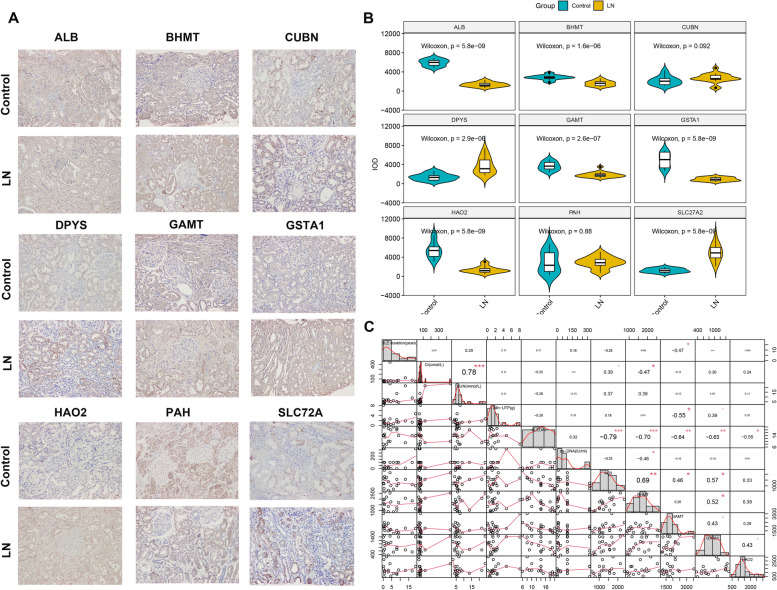
External validation of the alterations in model genes based on clinical specimens. **A** Representative immunohistochemistry images of the 9 model genes including ALB, BHMT, CUBN, DPYS, GAMT, GSTA1, HAO2, PAH, and SLC27A2. **B** Violin plots exhibiting the quantitative results of 9 model genes expression between controland LN patients. **C** Correlation analysis between the clinical characteristics and the expression of ALB, BHMT, GAMT, GSTA1, and HAO2 in LN glomerular tissues

Subsequently, we further analyzed the expression of 9-gene signature between control and LN glomerular tissues. The clinical information of 13 control and 19 LN patients were presented in Table 1. The immunohistochemistry images of ALB, BHMT, CUBN, DPYS, GAMT, GSTA1, HAO2, PAH, SLC27A2 between control and LN glomerular tissues were shown in Fig. 10A. Among them, the glomerular tissues from the LN group exhibited an increased integrated option density (IOD) of CUBN, DPYS, PAH, and SLC27A2. However, the IOD of ALB, BHMT, GAMT, GSTA1, and HAO2 was markedly curtailed in LN tissues when compared with the control group (Fig. 10B). These five down-regulated genes (ALB, BHMT, GAMT, GSTA1, and HAO2) were consistent with our above results. We therefore further evaluated the correlation between these genes and the clinical traits of LN patients. Interestingly, all of the genes exhibited a significant negative correlation with SLE scores (SLEDAI), suggesting the critical role of these five genes in alleviating the progression of SLE. In addition, BHMT was negatively correlated with creatinine (Cr) and ds-DNA, respectively, and GSTA1 was negatively correlated with 24 h urine protein quantification (24 h-UTP) (Fig. 10B), indicating that high expression of GSTA1 and BHMT may serve as protective factors for renal function.

**Table 1:** Basic information of controland LN patients in clinical specimens

	**Control (*N* = 13)**	**LN** **(*N* = 19)**	**Overall (*N* = 32)**
**Age**			
Mean (SD)	55.8 (9.47)	35.3 (11.8)	43.6 (14.9)
Median [Min, Max]	55.0 [39.0, 69.0]	34.0 [16.0, 55.0]	44.0 [16.0, 69.0]
**Sex**			
Female	4 (30.8%)	15 (78.9%)	19 (59.4%)
Male	9 (69.2%)	4 (21.1%)	13 (40.6%)
**SLE duration(years)**			
Mean (SD)	NA (NA)	5.54 (5.66)	5.54 (5.66)
Median [Min, Max]	NA [NA, NA]	4.00 [0.100, 19.0]	4.00 [0.100, 19.0]
Missing	13 (100%)	0 (0%)	13 (40.6%)
**Cr(umol/L)**			
Mean (SD)	77.3 (22.1)	82.3 (90.4)	80.3 (70.3)
Median [Min, Max]	73.0 [49.0, 139]	63.0 [36.0, 448]	65.0 [36.0, 448]
**BUN(mmol/L)**			
Mean (SD)	4.85 (0.802)	7.81 (5.74)	6.61 (4.64)
Median [Min, Max]	4.40 [3.90, 6.50]	5.70 [2.90, 23.8]	5.40 [2.90, 23.8]
**24 h-UTP(g)**			
Mean (SD)	NA (NA)	1.86 (1.85)	1.86 (1.85)
Median [Min, Max]	NA [NA, NA]	1.40 [0.0920, 8.10]	1.40 [0.0920, 8.10]
Missing	13 (100%)	0 (0%)	13 (40.6%)
**SLEDAI**			
Mean (SD)	NA (NA)	13.8 (4.40)	13.8 (4.40)
Median [Min, Max]	NA [NA, NA]	14.0 [6.00, 20.0]	14.0 [6.00, 20.0]
Missing	13 (100%)	0 (0%)	13 (40.6%)
**ds-DNA(IU/ml)**			
Mean (SD)	NA (NA)	92.8 (110)	92.8 (110)
Median [Min, Max]	NA [NA, NA]	100 [0, 320]	100 [0, 320]
Missing	13 (100%)	0 (0%)	13 (40.6%)
**C3(g/L)**			
Mean (SD)	NA (NA)	0.530 (0.337)	0.530 (0.337)
Median [Min, Max]	NA [NA, NA]	0.412 [0.170, 1.34]	0.412 [0.170, 1.34]
Missing	13 (100%)	0 (0%)	13 (40.6%)
**C4(g/L)**			
Mean (SD)	NA (NA)	0.0955 (0.0884)	0.0955 (0.0884)
Median [Min, Max]	NA [NA, NA]	0.0750 [0.0160, 0.372]	0.0750 [0.0160, 0.372]
Missing	13 (100%)	0 (0%)	13 (40.6%)

## Discussion

The diversity of pathophysiological and clinical manifestations leads to a poor prognosis in patients with LN. In addition, untimely diagnosis and treatment at the early stage also serve as critical factors contributing to the deterioration of LN. Therefore, it is urgent to screen more accurate molecular subtypes and establish appropriate diagnostic models for early identification and guidance of individualized treatment of LN.

Ferroptosis is a newly reported Fe^2+^-dependent cell death mode evidenced by lipid peroxide overload caused by intracellular oxidative stress [21]. It has been reported that several major pathways are currently involved in the regulation of ferroptosis. First, inhibition of the Xc^−^system has been shown to disrupt GSH and GPX4 synthesis by inhibiting cysteine uptake, lowering the activity of antioxidant products and increasing the expression of lipid peroxides, eventually leading to ferroptosis [22, 23]. Additionally, signaling pathways related to ferritin metabolism, such as the ATG5-ATG7-NCOA4and p62-Keap1-NRF2 axis, are thought to act as critical mechanism in regulating intracellular Fe^2+^ metabolisms and ROS formation, which are closely associated with ferroptosis [24, 25]. Moreover, phosphatidylethanolamine (PE) and polyunsaturated fatty acid (PUFA) generated from ACSL4 and p53-SAT1-ALOX15-dependent lipid pathways are another primary factors contributing to the activation of ferroptosis [26, 27]. A growing body of research demonstrates that ferroptosis has been implicated in multiple autoimmunity diseases, including SLE [28], multiple sclerosis [29] and cutaneous diseases [30]. For example, lower expression of intracellular GSH and GPX levels are associated with the severity of SLE patients, whereas reversal of GSH deletion alleviates the progression of lupus in mouse models [31, 32]. Recent studies found the activation of ferroptosis is also considered as the basis of chronic kidney diseases, including LN [14]. Bioinformatics analysis based on microarray datasets indicates that ferroptosis-related genes were closely related to the onset of LN [13]. However, the latest study only disclosed the relationship between ferroptosis-related genes and LN, the potential molecular mechanisms by which ferroptosis regulates LN progression still remain obscure.

In this study, we first systematically evaluated the expression profiles of FRGs between LN and control glomerular tissues and identified 10 differentially expressed FRGs in LN patients, most of which presented distinct interactions, indicating that ferroptosis may exert a critical role in the progression of LN. Immune infiltrating analysis suggested that the proportions of immune cells were markedly distinct between controland LN glomerular tissues as evidenced by a greater abundances of naïve B cells, plasma cells, activated NK cells, Monocytes, M2 macrophages and resting dendritic cells in patients with LN, which were consistent with the previous studies [33–37]. Correlation analysis revealed that CD8^+^ T cell, resting memory CD4^+^ T cells, Tregs, resting NK cells, Monocytes, and resting mast cells had the most apparent correlation with ferroptosis DEGs, indicating that the interaction of FRGs with immune cells may be the potential pathological mechanism leading to the onset and progression of LN. Subsequently, two distinct molecular subtypes of LN glomerular tissues were determined on the basis of the expression profiles of 10 differentially expressed FRGs.

Interestingly, LN tubulointerstitium tissues were also classified into two molecular subtypes, demonstrating the repeatability of the clustering. It has been widely recognized that the dysfunction of immune cells is a notable hallmark of LN [34, 38]. Nevertheless, whether the mechanisms underlying the poor prognosis in LN patients are closely associated with the differences in the immune cell ratios needs further elucidation. Therefore, we next evaluated immunological features between two distinct molecular subtypes and found Subtype 2 was primarily enriched in cytokine receptor interaction, immune-related pathways. Additionally, Subtype 2 exhibited significantly higher infiltration levels of memory B cells, M0 macrophages, and neutrophils, indicating that ferroptosis might be associated with the dysfunction of both innate immunity and adaptive immunity. Moreover, immune scores and immune checkpoints, including ICOS, CTLA4, CD86, CD70, CD40, CD27, and PDCD1 also exhibited higher levels in ferroptosis Subtype 2 compared to Subtype 1. Combined with these results, we can infer that ferroptosis Subtype 2 may efficiently activate immunity response and may benefit from immunotherapy.

Subsequently, we established a 9-gene protective model based on neural network machine learning for predicting the progression of LN, composed of ALB, BHMT, CUBN, DPYS, GAMT, GSTA1, HAO2, PAH, and SLC27A2. A scoring model (NeuraLN) exhibited a high AUC and P-R value in the validation set. More importantly, patients with Subtype 2 displayed higher NeuraLN when compared to patients with Subtype 1. In addition, higher immune infiltrating levels and immune scores were observed in high-NeuraLN patients. At the same time, correlation analysis revealed that NeuraLN was significantly correlated with memory B cells, CD8 + T cell, and M0 macrophages. These results demonstrated that the NeuraLN is a crucial indicator of progress in LN patients.

It is worth mentioning that we performed the clinical specimen validation and found five down-regulated genes, including ALB, BHMT, GAMT, GSTA1, and HAO2, which were consistent with the above bioinformatics analysis findings. Serum albumin (ALB) is a nutritional indicator that could largely reflect the living conditions of patients [39]. The decreased level of ALB has been proven to be associated with the severity of kidney diseases [40, 41].Another protective gene BHMT is a novel prognostic biomarker for various diseases. The BHMT-dependent methylation pathway, for example, has been shown to play a role in regulating oligodendrocyte maturation [42]. Deficiency or inhibitation of BHMT may be the vital factor leading to poor prognosis of hepatocellular carcinoma [43]. The creatine biosynthetic pathway is crucial for phosphate-related cellular energy production and storage, especially in tissues with high metabolic demands [44]. GAMT is a key enzyme involving the endogenous pathway of creatine biosynthesis and is more expressed in the liver and kidney [45]. Furthermore, glutathione transferase A1 (GSTA1) is a phase II conjugating enzyme that detoxifies electrophilic compounds such as carcinogens, therapeutics, environmental toxins, and oxidative stress products [46]. Recent studies have demonstrated that GSTA1-mediated ROS and Ca^2+^ signaling pathways are mainly implicated in the enhancement of aldosterone secretion, and low-activity of GSTA1 is associated with iron overload-induced kidney injury [47, 48]. Bioinformatics analysis suggests that increased HAO2 enable to promote lipid catabolism and prevent lipid accumulation, eventually delaying the progression of clear cell renal cell carcinoma [49]. These results were consistent with the findings in our current study that the down-regulation of ALB, BHMT, GAMT, GSTA1, and HAO2 may be predictive of poor prognosis in LN patients. Except for ALB, other genes are rarely reported in LN. In the current study, we identified these ferroptosis subtype-specific signature genes would as being capable of providing novel insights into the prognosis of LN.

Some shortcomings need to be emphasized in our study. Firstly, results based on existing databases would exhibit some deviation from reality due to confounding factors such as individual differences. Secondly, the clinical specimen for external validation is small, a larger and multi-center LN cohort need to be utilized for further research. Moreover, the underlying mechanisms of FRGs and ferroptosis-subtype related signature genes in LN require further exploration in more experiments. Though the results where connected to personalized medicine, a connection to current standard of care was not made.

## Conclusions

In summary, we systematically evaluated the expression of FRGs in LN and revealed an innovative molecular classification related to ferroptosis. We established a 9-gene protective model on the basis of neural network machine learning, which would be capable of accurately predicting the progression of LN patients. Importantly, external validation of clinical specimens confirmed that ALB, BHMT, GAMT, GSTA1, and HAO2 were associated with the prognosis of LN patients. The protective model based on multiple signature genes would provide a novel strategy to predict the progression of LN.

### Supplementary Information

Supplementary Material 1.

Supplementary Material 2.

Supplementary Material 3.

Supplementary Material 4.

## Data Availability

The datasets presented in this study can be found in online repositories. The names of the repository/repositories and accession number(s) can be found in the article/ supplementary material.

## Abbreviations

LN: Lupus nephritis
SLE: Systemic lupus erythematosus
FRGs: Ferroptosis-related genes
ESRD: End-stage renal disease
AKI: Acute kidney injury
CKD: Chronic kidney disease
IHC: Immunohistochemical
GEO: Gene Expression Omnibus
CDF: Cumulative distribution function
P-R: Precision-Recall
IOD: Integrated option density
DEGs: Differentially expressed genes
Tregs: Regulatory T cells
tSNE: T-Distributed Stochastic Neighbor Embedding
IOD: Integrated option density
Cr: Creatinine
PE: Phosphatidylethanolamine
PUFA: Polyunsaturated fatty acid
ALB: Serum albumin
GSTA1: Glutathione transferase A1
