# Supplementary figures and images for: Investigation of ferroptosis-associated molecular subtypes and immunological characteristics in lupus nephritis based on artificial neural network learning
Source: Arthritis Res Ther. 2024 Jul 3;26:126. doi: 10.1186/s13075-024-03356-z (PMC11220981; doi:10.1186/s13075-024-03356-z)

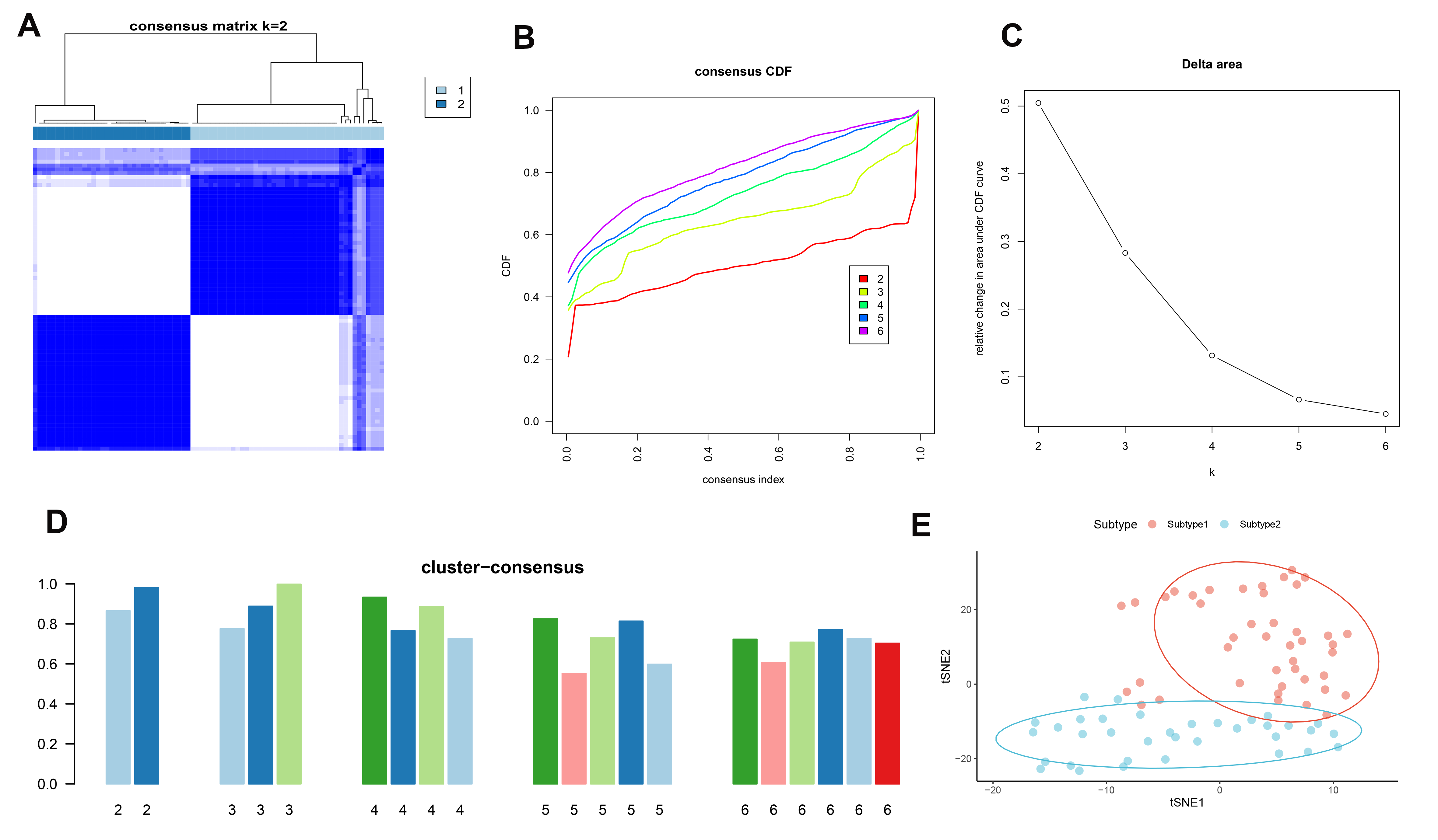

Supplement: Supplementary file 1 — Supplementary Material 1. [file 13075_2024_3356_MOESM1_ESM.tif]

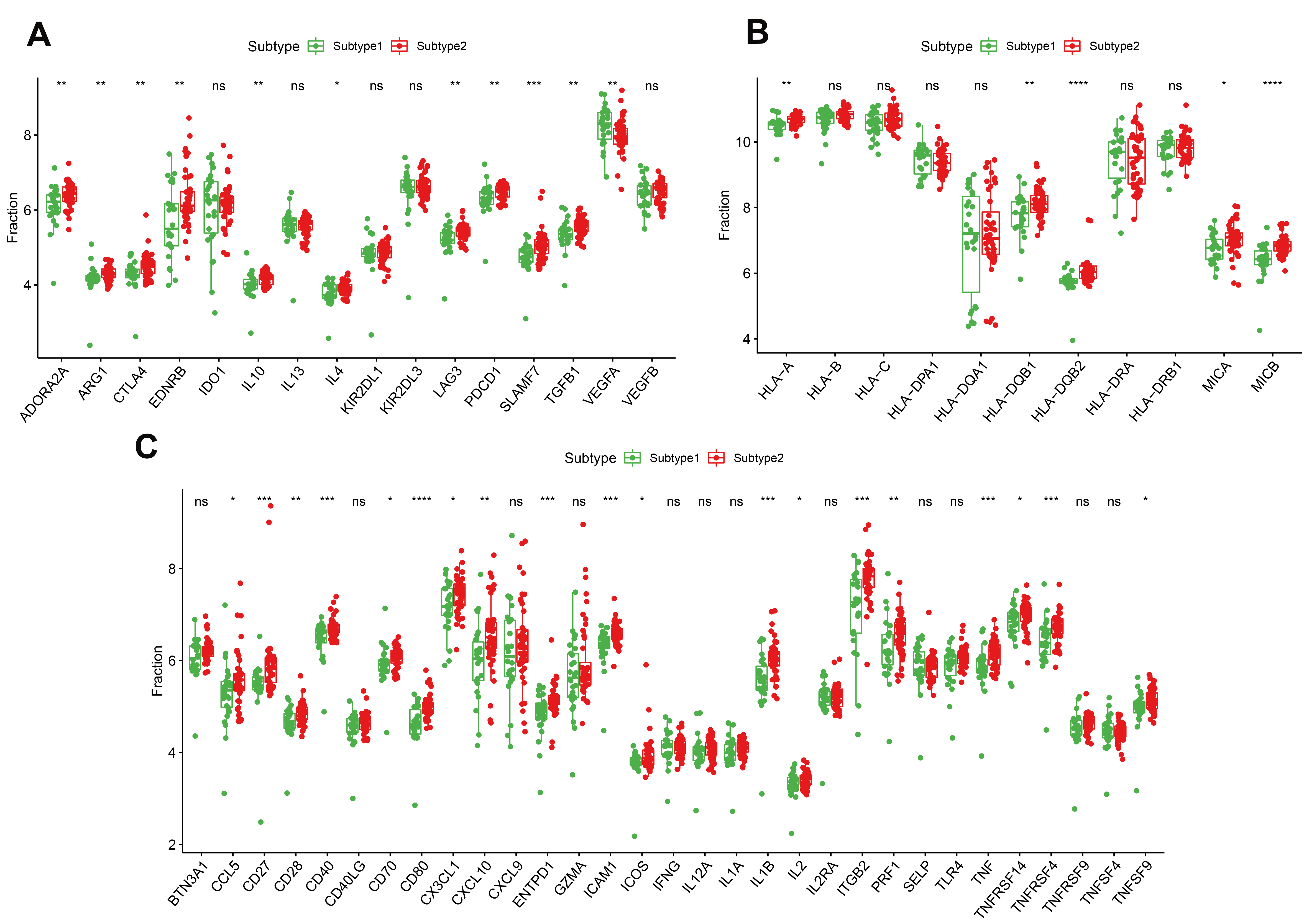

Supplement: Supplementary file 2 — Supplementary Material 2. [file 13075_2024_3356_MOESM2_ESM.tif]

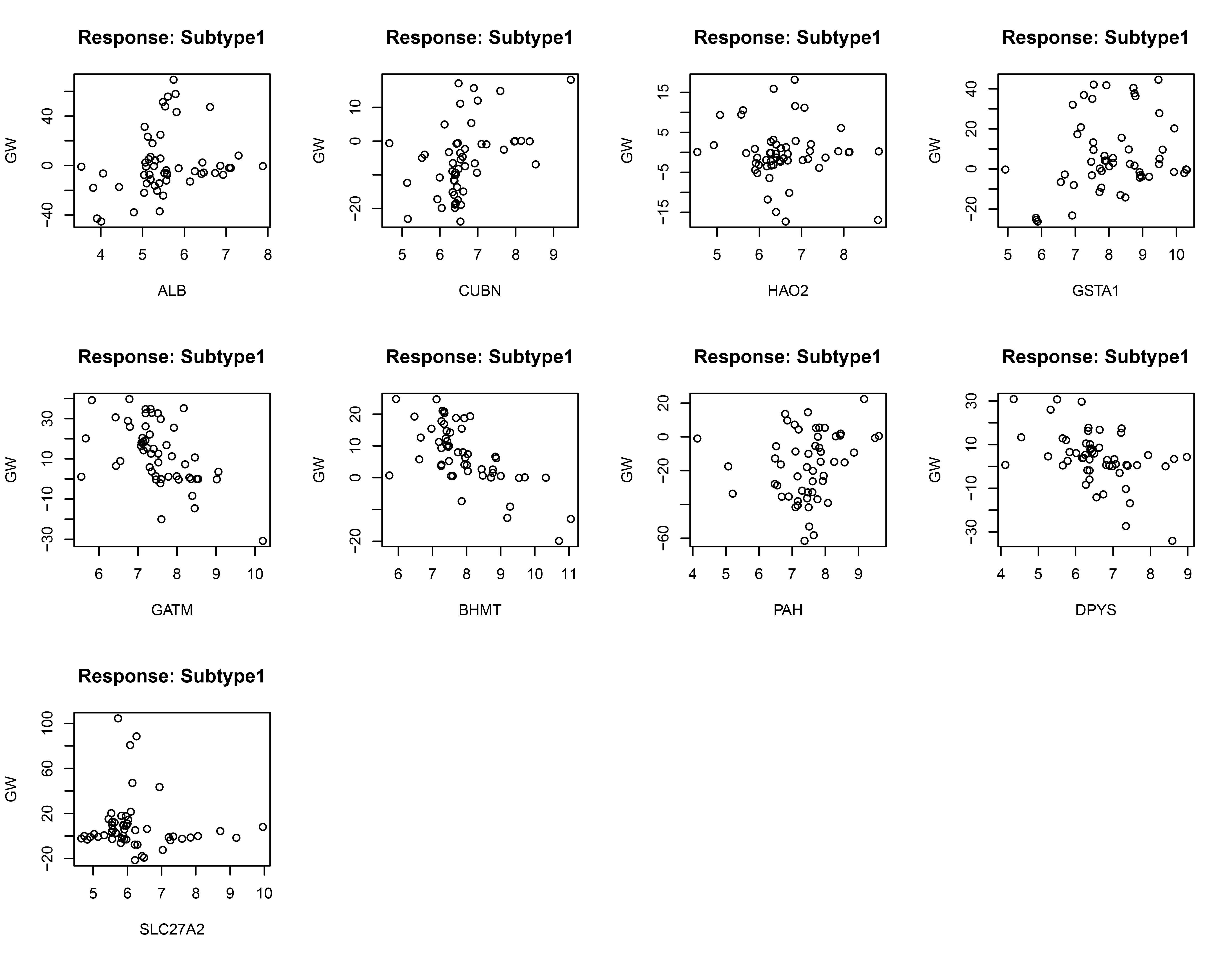

Supplement: Supplementary file 3 — Supplementary Material 3. [file 13075_2024_3356_MOESM3_ESM.tif]
